# Flank pseudohernia following posterior rib fracture: a case report

**DOI:** 10.1186/s13256-016-1054-9

**Published:** 2016-10-01

**Authors:** Adam M. Butensky, Leah P. Gruss, Zachary L. Gleit

**Affiliations:** 1 Columbia University College of Physicians and Surgeons, 5141 Broadway, Suite 3-170, New York, NY 10034 USA; 2 Department of Radiology, Columbia University College of Physicians and Surgeons, New York, NY USA; 3 Department of Surgery, Columbia University College of Physicians and Surgeons, 5141 Broadway, Suite 3-170, New York, NY 10034 USA

**Keywords:** Pseudohernia, Rib fracture, Hernia, Case report

## Abstract

**Background:**

A pseudohernia is an abdominal wall bulge that may be mistaken for a hernia but that lacks the disruption of the abdominal wall that characterizes a hernia. Thus, the natural history and treatment of this condition differ from those of a hernia. This is the first report of a pseudohernia due to cough-associated rib fracture.

**Case presentation:**

A case of pseudohernia due to fractures of the 10^th^ and 11^th^ ribs in a 68-year-old white woman is presented. The patient suffered from a major coughing episode 1 year prior to her presentation, after which she noted a progressively enlarging bulge in her left flank. Computed tomography demonstrated a bulge in the abdominal wall containing bowel and spleen but with all muscle and fascial layers intact; in addition, lateral 10^th^ rib and posterior 11^th^ rib fractures were noted.

**Conclusions:**

As there was no defect in muscle or fascia, we diagnosed a pseudohernia, likely due to a denervation injury from the fractured ribs. Symptomatic treatment was recommended, including wearing a corset and referral to a pain management clinic. Symptomatic treatment is thought to be the mainstay of therapy for pseudohernias, as surgical intervention is unlikely to be of benefit.

## Background

A pseudohernia is an abdominal wall bulge that resembles a hernia but differs from a true hernia in that there is no actual muscular disruption and all muscle and fascial layers remain intact. Pseudohernias are rare phenomena, which have been reported in association with a variety of syndromes involving neuropathy or denervation, including zoster infection [1], diabetes mellitus [2, 3], and following operative trauma [3–6]. The case presented describes a pseudohernia caused by rib fractures. A brief review of the literature concerning pseudohernias is presented for discussion.

## Case presentation

**Fig. 1:**
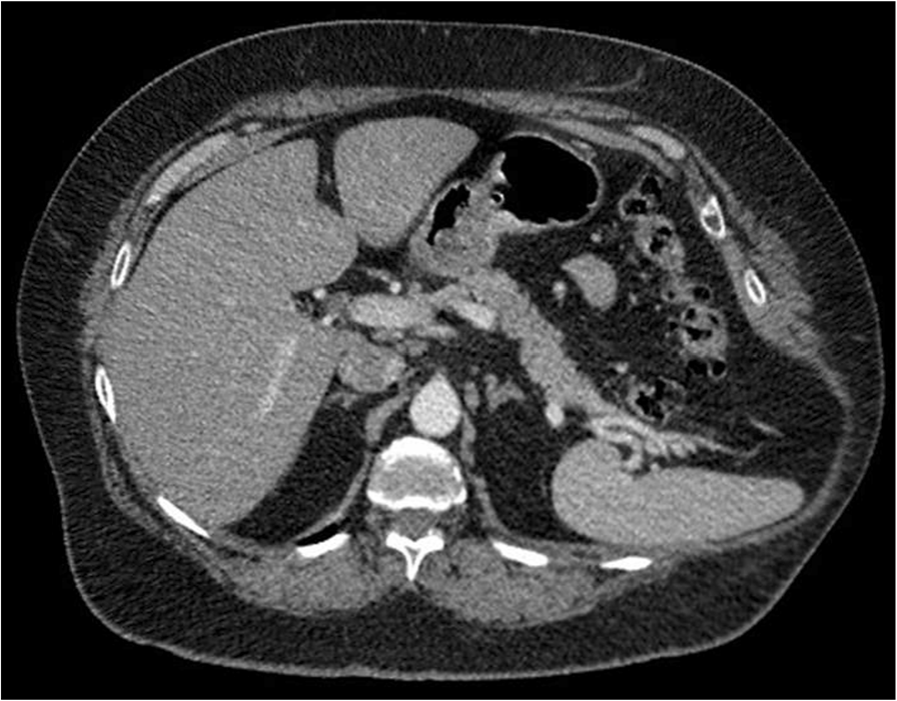
Axial contrast-enhanced computed tomography image. The image shows a bulge in the left posterolateral abdominal wall with fascial layers intact

**Fig. 2:**
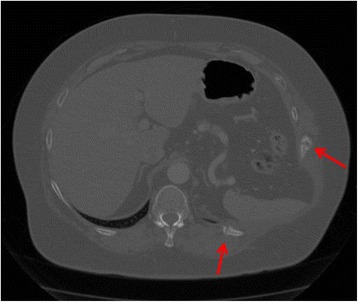
Axial computed tomography image displayed in a bone window. The image shows left lateral 10^th^ and left posterior 11^th^ rib fractures (*arrows*)

**Fig. 3:**
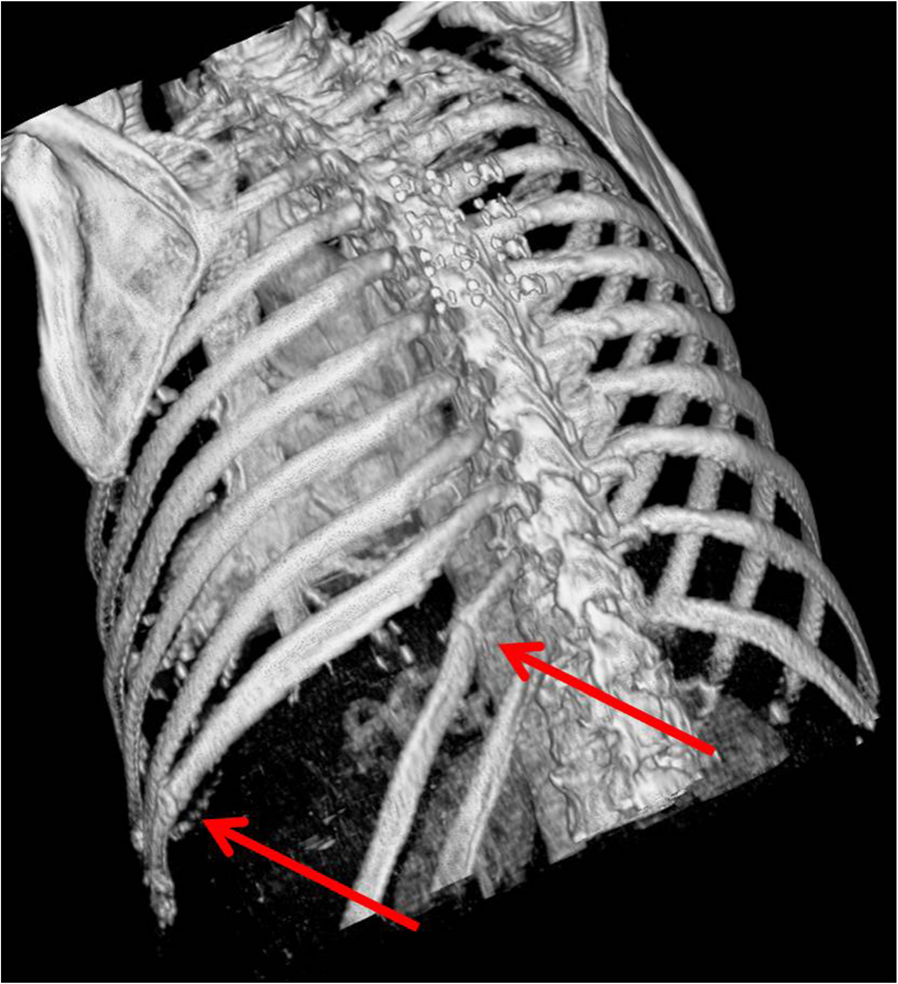
Three-dimensional reconstruction computed tomography image. The image shows left 10^th^ and 11^th^ rib fractures with splaying of the ribs secondary to underlying pseudohernia bulge (*arrows*)

A 68-year-old white woman with a past medical history of chronic obstructive pulmonary disease (COPD) presented to the clinic complaining of a bulge in her left flank that had progressed over the prior year. One year prior to presentation, she suffered a “coughing jag,” after which she developed pain in her left flank as well as the progressive flank bulge. The pain was constant, had a burning quality, and was intermittently severe. She denied a history of zoster or other neuropathy syndromes. Prior surgical history included a hysterectomy and an umbilical hernia repair. A computed tomography (CT) scan (Fig. 1) was initially read as showing a 10-cm hernia containing large bowel and spleen, with concomitant fractures of the lateral aspect of her 10^th^ rib and the posterior aspect of her left 11^th^ rib (Figs. 2 and 3). However, upon further review, the CT scan did not appear to demonstrate a defect in the musculofascial layers of the abdominal wall. As such it could be more aptly described as a pseudohernia, which we attribute to denervation related to the rib fractures.

Recommendations included wearing a corset to manage the bulge, weight loss, and evaluation in a chronic pain clinic to manage the pain associated with her pseudohernia, which was thought to be more likely related to nerve injury rather than to the bulge itself. Surgery was not felt to be indicated as this was not, in fact, a true hernia.

## Discussion

The phenomenon of pseudohernia was first described in 1936 by Loewe in the context of local anesthetic injection into the abdominal muscles of guinea pigs [7]. He noted an induced relaxation of the abdominal wall without any discontinuity in the abdominal wall musculature and termed the resultant bulge a “pseudohernia.” Loewe postulated that the pseudohernia was related to a sensory, rather than motor, neuron defect and that the bulge resulted from an interruption in the reflex arc that maintained abdominal wall tension. Pseudohernias have been reported in the literature in a variety of clinical contexts since Loewe’s initial description.

Pseudohernias have been described as sequelae of herpes zoster infection. A 2013 literature review analyzed 36 cases of pseudohernias related to segmental zoster abdominal paresis [1]. In 32 of these patients, the dermatomal rash characteristic of zoster preceded the onset of the pseudohernia. Interestingly, the most commonly affected nerves were T10-T12, which is consistent with the area of injury in the patient we describe here. Of the 29 patients with subsequent follow-up, 23 had complete recovery with a mean recovery time of 4.9 months.

Pseudohernias have also been described as sequelae of diabetic neuropathy. A 1999 case report described a 63-year-old man who experienced a pseudohernia 6 months following the onset of stinging left-sided abdominal pain and was found to have denervation of the left paraspinal muscles in the distribution of the T6-T10 myotomes on electromyography (EMG) [2]. His pseudohernia persisted for 1 year before gradually resolving. A 2002 Israeli case series described a similar case in a 62-year-old man, with resolution of the pseudohernia in 3 months [3].

Postoperative pseudohernias have been noted to occur following procedures involving flank incisions. A 2004 study of 70 patients who underwent radical or partial nephrectomy with flank or thoracoabdominal incisions found that 34 (49 %) of the patients reported durable, often painful, flank bulges after 1 year, thought not to be true hernias. Interestingly, flank bulges were more common on the left side, and the authors speculated that these bulges were related to denervation injuries, though they did not use the term “pseudohernia” [4]. Similarly, a 1994 study of patients following abdominal aortic aneurysm repair with retroperitoneal incisions found that when the incision extended into the 11^th^ intercostal space, 6/31 patients developed pseudohernias postoperatively [5]. Other reports have described pseudohernia following T9-T10 meningioma excision and following thoracoscopy [6].

Finally, two case reports describe “cough-induced intercostal hernias” associated with rib fractures, attributed to rupture of intercostal muscles [8, 9]. Both case reports described clinical and imaging findings very similar to those exhibited by our patient. No disruption of muscular layers was demonstrated on the images presented in these reports. Hence, we postulate that these cases may in fact have actually represented pseudohernias.

In summary, pseudohernia should be considered in the evaluation of flank or abdominal protrusions, particularly when a cause for denervation injury can be ascertained. Causes may be infectious, metabolic, iatrogenic, or traumatic. A CT scan can be a useful imaging modality and demonstrates an abdominal wall bulge with preserved integrity of the musculofascial layers of the abdominal wall, in contradistinction to a true hernia, in which a muscular or fascial disruption should be seen. It has been suggested that EMG may have utility to confirm the diagnosis. As there is no physical defect in the abdominal wall to be corrected, surgical intervention is not generally thought to be efficacious for the treatment of pseudohernias [3], though a successful laparoscopic mesh repair has been described [10]. More conservative measures, including mechanical support with a corset, adequate analgesia, physical therapy, and possibly evaluation in a pain treatment center may offer patients symptomatic improvement [6]. Regression of pseudohernias has been reported with resolution of the neuropathic etiology. However, when the nerve injury is permanent, pseudohernias and related pain are likely to persist.

## Conclusions

Pseudohernias are functional abdominal protrusions that resemble hernias but are not associated with evidence of muscular or fascial disruption. They are associated with neuropathy or neural disruption. In the case presented here, a pseudohernia resulted from an apparent nerve injury related to cough-induced rib fracture. Symptomatic relief, rather than surgical repair, is thought to be the mainstay of therapy for pseudohernias.

## Abbreviations

COPD: chronic obstructive pulmonary disease
CT: computed tomography
EMG: electromyography
